# Incidence of lung cancer among healthcare workers in Hunan Province and analysis of related risk factors

**DOI:** 10.3389/fpubh.2023.1280316

**Published:** 2024-01-05

**Authors:** Jinzhang Xiao, Sishi Li, Shudong Zhu, Kamakshi Ranjan, Dianzheng Zhang

**Affiliations:** ^1^Nantong Tumor Hospital, Nantong, Jiangsu, China; ^2^School of Medicine, Central South University, Changsha, China; ^3^School of Medicine, Nantong University, Nantong, Jiangsu, China; ^4^Argus Pharmaceuticals, Changsha, China; ^5^Department of Bio-Medical Sciences, Philadelphia College of Osteopathic Medicine, Philadelphia, PA, United States

**Keywords:** lung cancer, healthcare worker, risk factor, incidence, occupation

## Abstract

**Background:**

The prevalence of lung cancer, a major type of malignant tumor, has been increasing over the years greatly impacting the health of Chinese residents. This study investigates the epidemiological characteristics of lung cancer among healthcare workers in the Hunan Province, as well as the occupational risk factors.

**Methods:**

The data analyzed in this study was collected from the largest tumor hospital in the province: the Hunan Provincial Tumor Hospital affiliated with Central South University, School of Medicine. The data collected encompasses input collected between the years of 2004 to 2013 of the population of healthcare workers who were hospitalized for lung cancer treatments. Information was obtained through statistical analysis and telephonic interviews.

**Results:**

The prevalence of lung cancer among healthcare workers was much higher than that of the general population, as revealed by the difference between number of healthcare worker cases per 1,000 cases and number of healthcare workers per 1,000 population in the decade from 2004 to 2013. Analysis of the data further demonstrates that lung cancer prevalence among healthcare workers increases exponentially with age. Although smoking has been shown to increase the incidence of lung cancer to some extent, it is most likely not the main cause of lung cancer. In addition, it appears that the highest rates of lung cancer incidence occurs in mainly in primary general practitioners, medical radiologists, and nurses. The lack of awareness of personal safety measures may place healthcare workers at a greater risk of lung cancer.

## Introduction

In 2013, there were approximately 3.682 million new cases of malignant tumors nationwide, and 2.229 million deaths (1). The morbidity rate of malignant tumors is 190.17/100,000 and the mortality rate is 109.95/100,000 in the Chinese population (1), indicating malignant tumors are a great threat to human health and society. The incidence of malignant tumors is predominantly concentrated among individuals aged over 45 years. Lung cancer ranks as the most common cancer among males and the second most among women (1). Lung cancer is also the leading cause of death in both males and females (1). Between 2007 and 2013, lung cancer has also been the leading cause of both morbidity and mortality in China (1–7). Within the span of 3 years, between 2010 and 2013, the number of new cases of lung cancer in China increased by 133,000 reaching a total of 733,000 cases. Simultaneously, in 2013, the incidence of lung cancer-related deaths reached 591,000, due to a surge in an additional 101,000 deaths between 2010 to 2013 (1–7). These alarming trends underscore the importance of lung cancer study in regional and global health.

Healthcare workers are a broad group of professions including practicing doctors, physician assistants, registered nurses, pharmacists, laboratory technologists, imaging technologists, health supervisors, and trainee doctors. Due to the nature and setting of their work, healthcare workers are more likely to be exposed to various carcinogens that can adversely affect the lung than the general population.

### Formaldehyde

Formalin (containing 35–40% formaldehyde) is widely used in the fixation and preservation of specimens, particularly in the preparation and preservation of human medical specimens (8). Formaldehyde has a very low boiling point at 19 °C causing it easily evaporate at room temperature. The International Agency for Research on Cancer (IARC) has classified formaldehyde as a Group 1 human carcinogen, primarily associated with nasopharyngeal carcinoma but also potentially associated with lung cancer and leukemia (8). The carcinogenicity of formaldehyde is primarily attributed to its ability to fragment DNA and cross-link DNA and protein structures (9–12). Moreover, formaldehyde, as a potent oxidant that can generate reactive oxygen radicals resulting in cellular and tissue damage (13–15). Research by Liu et al. demonstrated a higher rate of tumor death in groups exposed to formaldehyde in comparison to groups not exposed to formaldehyde, in a formaldehyde plant (16). Cui et al. showed an increased incidence of malignant tumors, especially gastric cancer, liver cancer, and lung cancer among formaldehyde plant workers (17). Worldwide studies had also reported elevated cancer mortality among workers with industrial formaldehyde exposure (18, 19). A recent systematic evaluation exhibits that various well-designed, high-quality studies also support the association between the lung cancer risk and formaldehyde exposure (20). In a hospital setting, healthcare workers, particularly those working in dissection and pathology rooms, are at risk of formaldehyde exposure during specimen preparation and preservation.

### Radiation

Radiation exposure in hospitals primarily arises from tumor radiation therapy and X-ray procedures conducted in the laboratory department. Radiation causes DNA damage and genetic alterations (21). While fast growing tumor cells are more susceptible to radiation, normal cells may also accumulate DNA damages. Prolonged low-dose radiation exposure, spanning many years or even decades, may result in skin cancer, lung cancer, leukemia, and bone cancer (22). During radiation therapy or diagnosis, patients are often administered treatment by medical professionals, including doctors and nurses, who also might be exposed to radiation.

### Anticancer drugs (ADs)

Anticancer drugs, particularly cytotoxic drugs, form the backbone of cancer treatment. These drugs, while effective against tumors, can also impact on normal cells. Patients undergoing anticancer drug treatment often experience various adverse reactions, with myelosuppression and gastrointestinal issues being the most common (23). Additional side effects include alopecia, liver toxicity, lung toxicity, kidney toxicity, and neurotoxicity. Long-term exposure to anticancer drugs, especially for healthcare workers such as oncology nurses, may be detrimental to health. Studies have indicated that anticancer drugs can be detected in the air during the preparation and administration of these drugs (24–27). The turbulence generated during preparation can lead to the formation of aerosols, allowing drug particles to enter human bodies through the respiratory tract and skin (28–30). Several studies have detected the presence of anticancer drug molecules in the urine of healthcare workers exposed to these drugs (31, 32). These compounds can harm the immune system, disrupt hormone secretion, damage DNA, and even lead to cancer (33). Many general hospitals and chemotherapy units lack comprehensive protective equipment and do not supply sufficient safety education. Nurses in China, in particular, often do not prioritize their own safety (34). A survey of nurses in 78 city and county-level hospitals in the southwestern provinces in China revealed gaps in awareness and protection norms (35).

### Bodily secretion

Healthcare workers often encounter bodily secretions including vomit, excrement, and saliva, in hospital environments where patients are closely confined. These professionals are exposed to a dense population with a diverse range of patients leading them to be at a higher risk of contracting an infection compared to the general population. Studies have identified a higher risk of tuberculosis (TB) among healthcare workers in respiratory departments. Among the different health professionals, doctors working in departments related to the respiratory tract are at an even greater risk than others (36). Healthcare workers are found to be 10–20 times more likely to contract TB in hospital settings compared to the general population. Research in Tianjin indicated that a significant portion of medical staff did not consistently wear masks when in contact with suspected TB patients, contributing to increased risk of exposure to the infection (37). The coexistence of pulmonary tuberculosis and lung cancer is very common with studies indicating an increased risk of lung cancer among TB patients (38–40).

Other risk factors such as clinical trauma due sharp instrument injury or needle stab injury are also common in the medical practice of healthcare workers. The infection after clinical trauma may transmit infectious diseases like TB and viral infection, which may potentially be cancerous.

In summary, occupational hazards including exposure to formaldehyde, radiation, anticancer drugs, and infectious diseases, may place healthcare workers at significant risk for negative health outcomes. Determining whether the health of healthcare workers is indeed affected and the extent to which it is affected is the objective of this study. Addressing these risks and therefore promoting corresponding safety measures will protect the well-being of healthcare professionals and reduce the incidence of occupational diseases such as lung cancer. We report herewith that the prevalence of lung cancer among healthcare workers is much higher than that of the general population. Increasing awareness and compliance of proper safety precautions and protocols will reduce the occupational risks healthcare workers face and ultimately improve their health outcomes.

## Methods

This study collected data of lung cancer patients who were hospitalized in the Thoracic Surgery Department of the Hunan Provincial Tumor Hospital Affiliated to School of Medicine, Central South University between 2004 and 2013. The study collected personal information of the patients who were healthcare workers, including gender, age, occupation, home address, telephone number, discharge records, past medical history, marriage and childbirth history, and family history. A total of 16,514 cases were reviewed, including 237 cases of healthcare workers.

**Figure 1:**
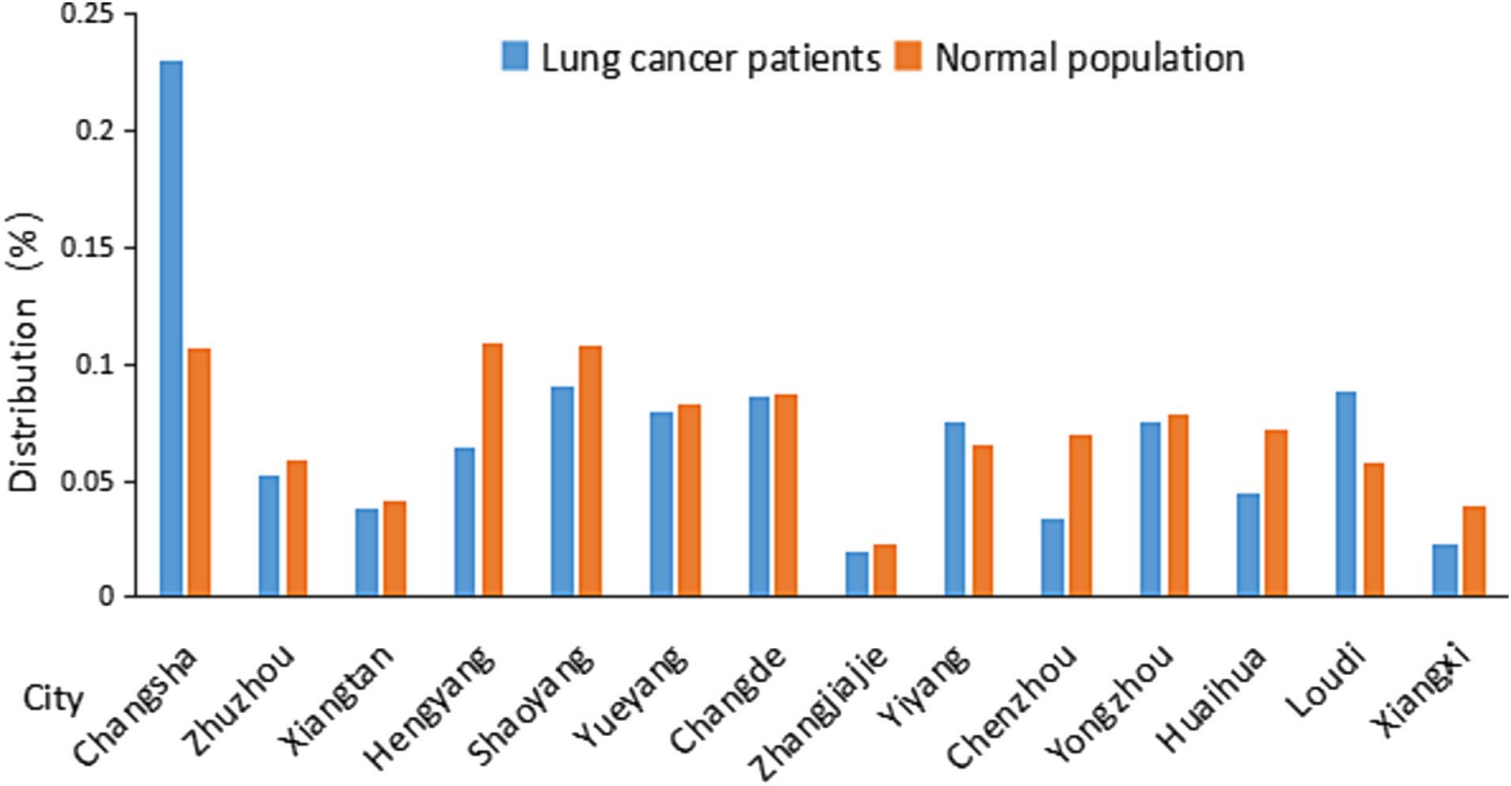
Distribution of lung cancer patients and normal population in Hunan Province in different cities. Distribution data were from Table 1.

The home address information of the 16,514 cases were collected and assigned to each city according to the administrative division of Hunan Province (Table 1; Figure 1). The number of healthcare workers per 1,000 lung cancer patients and the number of healthcare workers per 1,000 normal population were calculated and compared (Table 2). Statistical analysis was performed using paired, one-tailed *t* test.

**Table 1:** 2006–2013 distribution of lung cancer patients in Hunan Province in different cities.

City	Cases	LCP (%)	NP (%)*
All Hunan Cities	13,324	100	100
Changsha	3,072	23.06	10.72
Zhuzhou	694	5.21	5.87
Xiangtan	501	3.76	4.18
Hengyang	850	6.38	10.87
Shaoyang	1,203	9.03	10.77
Yueyang	1,067	8.01	8.34
Changde	1,151	8.64	8.70
Zhangjiajie	265	1.99	2.25
Yiyang	1,005	7.54	6.57
Chenzhou	450	3.38	6.98
Yongzhou	1,000	7.51	7.89
Huaihua	588	4.41	7.22
Loudi	1,180	8.86	5.76
Xiangxi	297	2.23	3.88

LCP (%): Percentage of lung cancer patients of each city in the province. NP (%): Percentage of population of each city in the province. ^*^From the Sixth National Population Census of Hunan Province.

**Table 2:** 2004–2013 the number of healthcare workers per 1,000 lung cancer patients and the number of healthcare workers per 1,000 normal population.

Year	2004	2005	2006	2007	2008	2009	2010	2011	2012	2013	Average	*p* value
Total	1,163	1,218	1,048	1,058	1,259	1,428	1717	2,207	2,405	2,793		
Case*	11	19	22	19	17	21	25	28	32	43		
A**	3.15	3.18	3	3.21	3.34	3.62	3.81	3.96	4.47	4.56	3.63	
B***	9.46	15.6	20.99	17.96	13.5	14.71	14.56	12.73	13.05	15.41	14.80	
Ratio#	3.00	4.91	7.00	5.60	4.04	4.06	3.82	3.21	2.92	3.38	4.19	<0.001

^*^Number of lung cancer cases among healthcare workers. ^**^Number of healthcare workers per 1,000 population in Hunan Province, from 2005–2014 China Health Statistics Yearbook. ^***^Number of healthcare workers per 1,000 lung cancer cases in Hunan Province (number of cases of sick doctors/total number of cases×1,000). ^#^Ratio of B/A. *P* value: paired, one-tailed *t* test.

The study also conducted telephone interviews with the 237 cases of lung cancer patients who were healthcare workers. The survey consisted of a questionnaire on their occupation, working age, working time, and related occupational risk factors. The survey also assessed the duration and frequency of exposure to formalin, radiation, anticancer drugs, infectious secretions, risk factors involved in their scientific research, the number of workplace injuries and safety practices. Patients were also asked to describe/identify the likely cause of their lung cancer for verification. Data was collected on 57 patients (those patients who had died or refused to answer questions were not included). The occupations of the 57 patients were classified and counted. These are reflected in Tables 3–5.

**Table 3:** Age and consumption of cigarettes or alcohol by healthcare workers contracting lung cancer.

	Cases	LCP (%)	NP (%)	Ratio (LCP/NP)**
Total	237	100	100*	
Age				
0–25	0	0	8.7	0
25–34	2	0.8	35.6	0.023
35–44	17	7.2	29.2	0.247
45–54	42	17.7	17.3	1.023
55–59	43	18.2	5.2	3.5
60-	133	56.1	4.0	14.025
Smoking history				
None	112	47.3	75***	
Occasionally	1	0.4		
<20 year	0	0		
20 ~ 30 year	20	8.4		
30 ~ 40 year	48	20.3		
≥40 year	56	23.6		
Drinking alcohol				
never	164	69.2		
<Once/month	24	10.1		
≥Once/month	49	20.7		
Smoking intensity				
Never	112	47.3		
Occasionally	0	0		
<10/d	4	1.7	13.4****	12.7
10 ~ 20/d	9	3.8	28.7	13.3
20 ~ 30/d	64	27	35.6–58	46.6–75.8
≥30/d	48	20.2	<22.4	>90.2

*From “2013 China Health Statistics Yearbook.” **Ratio of percentage of lung cancer population (LCP, 3rd column) to that of normal population (NP, 4th column) of healthcare workers. ***References [41–43]. ****References [44].

**Table 4:** Occupational distribution of lung cancer patients among healthcare workers.

Occupation/Working years	Cases	LCP (%)	NP (%*)	Ratio (LCP/NP)
Total	57	100 (41cases**)	100****	100
General practice				
Total	12	29.3	7.6	3.875
0–30	0			
30–40	4			
40–50	8			
Internal medicine				
Total	9	22.0	32.5	0.676
0–30	2			
30–40	1			
40–50	6			
Surgery (Including orthopedics)				
Total	6	14.6	19.1	0.764
0–30	0			
30–40	4			
40–50	2			
Obstetrics & Gynecology				
Total	5	12.2	13.8	0.882
0–30	2			
30–40	1			
40–50	2			
Medical imaging				
Total	4	9.8	9.6	1.025
0–30	0			
30–40	2			
40–50	2			
Traditional Chinese medicine				
Total	5	12.2	17.4	0.701
0–30	0			
30–40	3			
40–50	2			
Total	57	100 (57cases***)		
Pharmacist				
Total	3	5.3		
0–30	2			
30–40	0			
40–50	1			
Nurse				
Total	9	15.8		
0–30	1			
30–40	7			
40–50	1			
Rural medical doctor				
Total	4	7.0		
0–30	0			
30–40	1			
40–50	3			

^*^Composition of subspecialty practicing physicians in the five-year health statistics yearbook from 2009 to 2014 excluding physicians practicing in village clinics. Since the composition has remained stable over the years, the representative composition of 2009 is listed for comparison. ^**^The numbers of village clinics, nurses and pharmacists (3 + 9 + 4 = 16) are not included in the total for side-by-side comparison of percentages (LCP% versus NP%). ^***^The proportion of pharmacists, nurses, and rural doctors in the total number of cases (57 cases) obtained in the follow-up visit. ^****^Information on the composition ratio of nurses, pharmacists, and rural doctors among the total healthcare workers was absent in the five-year health statistics yearbook. Current 100% including cases in General Practice, Internal Medicine, Surgery, Obstetrics & Gynecology, Medical Imaging, Traditional Chinese Medicine is only part (70.1%) of all departments (cases) of healthcare workers in the 2009 health statistics yearbook. Ratio (LCP/NP): Ratio of percentage of lung cancer population (LCP, 3^rd^ column) to that of normal population (NP, 4th column) of healthcare workers.

**Table 5:** Occupational risk factor: exposure information in lung cancer cases of healthcare workers.

Risk factor	Cases (57)
Radiation	
0 year	49
1–5 year	4
30–35 year	4
Trauma	
None or very few	52
Yes and more than a few	5
Anticancer drugs	
None or very few	56
Yes and more than a few	1
Exposure to secretions	
None or very few	37
Occasionally	7
Often	13
Self-protection awareness	
Low	13
General	10
High	23

## Results

### Regional distribution of lung cancer

Regions with a total of 13,965 cases were recorded at Hunan Provincial Tumor Hospital during the eight-year period spanning from 2006 to 2013. These data are organized in Table 1. Within this dataset, 415 cases were found to be missing, resulting in a missing rate of 3.00%. Furthermore, 227 cases were from other provinces, while only 13,324 cases originated from Hunan Province. Unfortunately, regional information regarding lung cancer patients for the years 2004 and 2005 is not available.

Table 1 and its associated Figure 1 shows that the lung cancer cases are relatively concentrated in the city of Changsha. There is a similar distribution of lung cancer to the distribution of normal population among most cities with the exception of Changsha.

### Prevalence of lung cancer in healthcare workers

Table 2 shows that the number of healthcare workers per 1,000 lung cancer cases within the decade of 2004–2013 was approximately 4 fold that of the number of healthcare workers per 1,000 normal population, which is statistically significant (*p* < 0.001). This implies that the probability of healthcare workers contracting lung cancer is much higher than that of the general population.

### Basic information of healthcare workers contracting lung cancer

**Figure 2:**
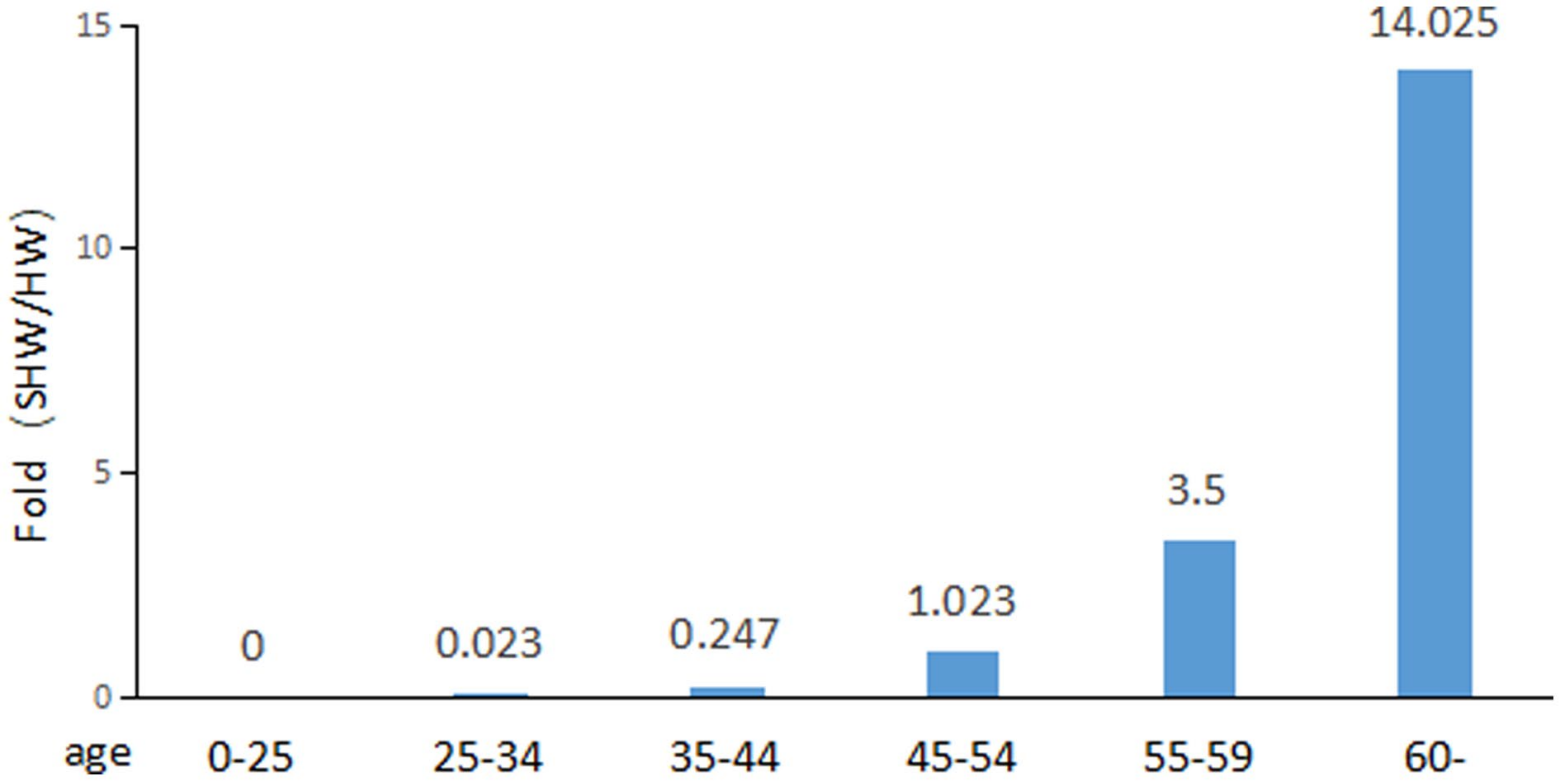
Fold change of percentage of sick healthcare workers (SHW) to that of healthcare workers (HW).

Table 3 shows that as age increases, the prevalence of lung cancer among healthcare workers also rises. In the 25–34 age group, healthcare workers account for 35.6% of the total number of healthcare workers, whereas in this age group, cancer-contracting healthcare workers account for only 0.8% of the total number of cancer-contracting healthcare workers, making the percentage of cancer-contracting healthcare workers 0.023 fold of that of healthcare workers (0.8%/35.6%) (Figure 2). In contrast, for those aged over 60, healthcare workers represent only 4% of the total number of healthcare workers of all ages, while cancer-contracting healthcare workers account for 56.1% of the total number of cancer-contracting healthcare workers of all ages, making the percentage of cancer-contracting healthcare workers 14.025 fold that of healthcare workers (56.1%/4%) (also see Figure 2). This stark increase from 0.023 to 14.025 indicates that lung cancer predominantly affects healthcare workers over the age of 60. The data obtained from all age groups also demonstrates an overall positive correlation between age and incidence of lung cancer (Figure 2).

Among the cancer-contracting healthcare workers, 52.7% (100–47.3%) have a history of smoking cigarettes (Table 3). In contrast, only 25% (100–75%) of all healthcare workers have a history of smoking cigarettes (Table 3). Moreover, the prevalence of lung cancer increases with the intensity of smoking, with over 20 cigarettes per day accounting for 47.2% (27.0% + 20.2%) of all cancer-contracting healthcare worker, in contrast to only 3.8, 1.7% or 0% in groups smoking 10–20, <10, and 0 cigarettes/day. This suggests that smoking may indeed influence the incidence of lung cancer among healthcare workers to an extent, although it is likely not the primary cause of lung cancer among this population.

Interestingly, the prevalence of lung cancer among healthcare workers appears to have little correlation with alcohol consumption.

### Occupational distribution of lung cancer patients

Follow-up visits of the 57 healthcare workers that were diagnosed with lung cancer (Table 4) reveals that lung cancer is most prevalent in Departments of General Practice (29.3%) and Interna Medicine (22.0%). Considering the relative low number of staff in the Departments of General Practice (7.6%) and Medical Imaging Departments (9.6%), it appears that workers in the Departments of General Practice and Medical Imaging Departments are most likely to contract lung cancer among all the healthcare workers, with the ratio of LCP/NP of 3.875 and 1.025, respectively (Table 4).

### Occupational risk factors

Questions and answers pertaining to formalin, radiation, anticancer drugs, exposure to saliva and other secretions, frequency and duration of exposure to risk factors in scientific research, the number of traumatic incidents, and adherence to safe guidelines are crucial aspects of our investigation. Due to the limited size of our dataset, we have not been able to compile a comprehensive table of occupational risk factors. However, Table 5 listed the most reliable records of risk factors involved in the follow-up visits. Table 5 shows that either of the exposure to radiation, traumatic incidents, anticancer drugs is not an important contributing factors to the development of lung cancer in the healthcare workers. However, a lack of self-protection awareness and exposure to saliva and other secretions may contribute to the increased incidence of lung cancer in healthcare workers.

In conclusion, our study found that the incidence of lung cancer in Changsha, the provincial capital, is significantly higher than the incidence in other cities. We also observed a consistently higher number of healthcare workers per 1,000 lung cancer cases in our study compared to the number of healthcare workers per 1,000 in the general population from 2004 to 2013. Additionally, age and smoking are associated with prevalence of lung cancer in healthcare workers. We have also characterized risks associated with occupational subspecialties, as well the potential exposure risk factors associated with the high incidence of lung cancer among healthcare workers.

## Discussion

It is interesting that lung cancer patients are relatively higher in Changsha than the other cities of Hunan Province (Figure 1; Table 1). Several factors may contribute to this observation: 1. Air Quality: Provincial capital cities, due to their larger populations, industrial activities, and traffic congestion, often experience poorer air quality. Changsha is one of the cities with heaviest air pollution in the middle triangle urban agglomerations (45). Research has shown a strong correlation between PM2.5 and the incidence rate of male lung cancer in urban areas. In urban areas, if PM2.5 levels change by 10 μg/m^3^, the shift in incidence rate relative to its mean increases significantly by 3.97% (95% CI: 2.18, 4.96%, *p* = 0.000) compared to rural areas (46). 2. Smoking: Smoking is a significant cause of lung cancer (3), and in many cases, capital cities have higher smoking rates due to various factors such as stress, lifestyle, and greater accessibility to tobacco products. 3. Healthcare Facilities: Provincial capitals often own better healthcare facilities, which might lead to higher rates of diagnosis and reporting of lung cancer cases.

Our study was focused on the healthcare workers in the province. It is surprising that the number of healthcare workers per 1,000 lung cancer cases in the decade 2004–2013 was approximately 4 fold that of the number of healthcare workers per 1,000 normal population. This stunning revelation implies that the chance of healthcare workers contracting lung cancer is much higher than that of the general population.

This survey underscores the significant relationship between lung cancer incidence with both age and smoking. It shows that when age is increased, or when the duration or intensity of smoking is increased, there is a notable rise in the incidence of lung cancer, suggesting DNA damage either accumulated naturally with age or caused by smoking may be a significant contributor to lung cancer morbidity (1–7). Notably, although not included in the results section, our survey also demonstrates that the male-to-female ratio among healthcare workers with lung cancer is 166:71; among male healthcare workers who had developed lung cancer, the ratio of smokers to non-smokers is 125:41, while only 0:71 for females. These extra lines of evidence are not only in consensus with the tumorigenic effects of smoking, but also indicates that smoking is not a prominent factor in the development of lung cancer among women. Indeed, our telephone interview survey revealed that most women patients and their families attributed their lung cancer to non-smoking risk factors such as exposure to (cooking) oil fumes and life stressors.

Occupationally, the highest prevalence of lung cancer is observed among general practitioners and medical imaging technicians. This pattern can be attributed to the substantial workload faced by professionals in these roles, along with prolonged patient interactions. Furthermore, grassroots hospitals where these professionals work often suffer from understaffing and inadequate equipment resources. Issues with accessibility along with a lack of understanding and indifference for self-protection and the use of personal protective equipment may contribute to this outcome. Importantly, there is a notable absence of a comprehensive occupational exposure reporting system within grassroots hospitals. An earlier survey by Zhu Lihong et al. in 2008 indicated that awareness of hand hygiene among medical staff was generally weak, with an implementation rate ranging from 50 to 70% (47). Additionally, radiologists are exposed to the causes of radiation emitted by imaging equipment (48).

Given the limited number of healthcare worker cases in this survey, it is also important to recognize the potential errors and limitations of our report. In conclusion, effective mitigation of occupational risks for healthcare workers requires collaboration among government bodies, medical units, and individuals. Governments should establish and enforce standardized regulations to address various occupational risk factors, while medical units should provide healthcare workers with comprehensive safety operation guidelines, a secure working environment, and ongoing safety awareness training. This collective effort is vital for safeguarding the health and well-being of healthcare professionals. Through this study, we hope to raise awareness of the additional risks healthcare workers face and encourage the implementation of protocols that can help decrease the disproportionately higher rate with which healthcare workers develop lung cancer.

## Data availability statement

The original contributions presented in the study are included in the article/supplementary material, further inquiries can be directed to the corresponding author.

## Ethics statement

Ethical review and approval was not required for the current study in accordance with the local legislation and institutional requirements. Written informed consent for participation was not required for this study in accordance with the national legislation and the institutional requirements.

## Author contributions

JX: Formal analysis, Writing – original draft, Data curation, Investigation, Methodology, Validation. SL: Formal analysis, Investigation, Methodology, Writing – original draft. SZ: Supervision, Writing – review & editing, Conceptualization, Funding acquisition. KR: Formal analysis, Writing – review & editing. DZ: Formal analysis, Writing – review & editing.
